# Robotic surgery training

**DOI:** 10.1590/1516-3180.2022.1415310823

**Published:** 2023-10-30

**Authors:** Pedro Henrique Xavier Nabuco de Araujo, Paulo Manuel Pêgo-Fernandes

**Affiliations:** I MD, PhD. Collaborating Professor of Thoracic Surgery, School of Medicine, Universidade de São Paulo (FMUSP), São Paulo, SP, Brazil.; II MD, PhD. Vice-director of the School of Medicine, Universidade de São Paulo (FMUSP), São Paulo, SP, Brazil; Full professor at the Department of Cardiopulmonology FMUSP, São Paulo (SP), Brazil. Director of the Scientific Department of the Associação Paulista de Medicina (APM), São Paulo, SP, Brazil.

Robot-assisted surgery emerged in the 2000s and has grown almost exponentially in the last decade. The use of robotic-assisted surgery has increased 10–40-fold more than that of laparoscopic surgery for general routine procedures.^1^ The continuous improvement of robotic platforms has allowed surgeons to overcome the limitations of conventional laparoscopy, such as 2D visualization and long instruments that do not accurately reproduce human wrist movements. Robotic systems provide high-definition 3D visualization, giving control of the camera to the surgeon. Robotic platforms have surgical instruments with intracavitary joints that reduce tremors, reproducing the movements of the surgeon on the console with great accuracy. Combined with these technical advantages, the clinical results consistently demonstrated in scientific articles seem to corroborate the great growth of robotic surgery in several specialties.^2^

The increasing use of robotic systems has raised concerns about the safety of patients operated on by surgeons on a learning curve. This demand resulted in a standardized curriculum for training new surgeons. A few years ago, robotic training was controlled and certified by Intuitive, the company that manufactured the only robotic platforms then available in Brazil. The significantly increased demand for robotic surgery led the Brazilian Medical Association (AMB), the Specialty Societies, and the Federal Council of Medicine (CFM) to regulate robotic surgery in Brazil, establishing a structured training curriculum^3,4^ consisting of a basic and an advanced stage. The basic or pre-clinical stage includes acquiring theoretical knowledge about robotic equipment and how the robot works, online training on the fundamentals of robotic surgery, watching videos and attending some robotic surgeries in person, training on a robotic simulator, and training on the robot console simulating real surgery movements and procedures (in-service training). In the advanced stage, the apprentice performs the robotic procedure as the main surgeon under the supervision of a surgeon-instructor (proctor) with extensive experience in the technique. After supervising at least ten specialty procedures, the proctor will be able to certify the apprentice surgeon’s competence to perform robotic surgery. Next, we will describe the stages of the structured curriculum in more detail.

## ONLINE TRAINING

The Fundamentals of Robotic Surgery (FRS) is an online program on the principles of robotic surgery developed by more than 80 experts. Actually, it is not just an online program because it included other training stages. It is divided into an introduction to the robotic surgical system, didactic instructions on robotic surgical systems, psychomotor skills curriculum and training, communication skills training, and staff training. However, the program that is currently mostly used is the Technology Training Pathway, developed by Intuitive Surgical for its platform. This website provides videos and documents about the principles of the da Vinci system, especially about robotic instruments and accessories, port placement, docking, intraoperative configuration, surgical console manipulation, troubleshooting, and safety resources.^5^

## BEDSIDE EXPERIENCE

This stage allows the apprentice surgeon to consolidate some of the concepts taught in the online stage. Watching and, ideally, participating in robotic surgeries in theater as a supervised assistant, the apprentice practices proper patient positioning, port placement, docking, and handling the robot’s arms and instruments, in addition to learning how to solve system problems. Live observation of an experienced surgeon allows the apprentice to become more familiar with the main standard procedures in their specialty and learn some critical points and technical tricks.^6^

## SIMULATION

The apprentice surgeon uses simulators to practice the skills required for robotic procedures. The exercises include handling articulated and 3D optic instruments, improving forceps movements, suturing, tying surgical knots, dissecting structures, and using different forms of energy. The most used 3D simulators are the dVSS da Vinci Simulator (3-D Systems/Simbionix), adapted to the surgeon’s console on the da Vinci platform, and the DV-Trainer (Mimic Technologies). In both models, the apprentice receives scores that evaluate different aspects of their movements throughout the exercises, helping to improve their weaker points.

In addition to virtual reality simulators, there are also physical simulators in which the surgeon performs exercises to develop skills such as suturing, cauterizing, and closing planes in organic tissues and anatomical parts. For an experience even closer to reality, animal organs such as porcine models can be used inside domes and manikins. However, due to their high costs, they are reserved for more advanced training stages or for the final evaluation of the pre-clinical part of robotic certification. A cadaveric human model is also a very realistic option, but it is very expensive.^6^

## SUPERVISED PROCEDURES

After being certified in the pre-clinical/basic stage, the apprentice surgeon can begin the clinical/advanced stage. This stage consists of at least ten robotic procedures supervised by an instructor surgeon with extensive robotics experience. Ideally, the first surgeries should be simpler procedures, and as the apprentice gains confidence with robotic technology, they move on to more complex procedures. When this progression is not possible, the apprentice can begin by receiving help from the instructor in the more technically difficult stages of complex procedures, gaining more autonomy as they gain experience. At the end of the advanced stage, the apprentice surgeon will be able to continue independently, as long as the proctor deems that they are ready to do so.

## SYSTEMS AVAILABLE IN BRAZIL

In Brazil, we currently have platforms produced by three companies. Most of the robotic systems are produced by Intuitive Surgical, the American company that pioneered robotics. The available platforms are the da VinciÒ Si, X, and Xi, with the Si model being discontinued over the next few years and gradually replaced by the VinciÒ X and Xi models. One hundred and three da VinciÒ systems are distributed in 88 hospitals across all Brazilian regions. The second company that came to Brazil was CMR Surgical, a British company that produces the VersiusÒ platform. At the moment, there are six VersiusÒ systems installed in six different hospitals in the South and Southeast regions. Finally, we have the HugoÒ RAS platform, produced by Medtronic, already available in two hospitals in São Paulo, SP. However, to date, the HugoÒ system is only validated for urological and gynecological surgeries.

The increased number of new platforms requires structured curriculum adaptations because even accredited surgeons will require training to use each of the different systems. Furthermore, an increasing number of surgeons will begin their experience directly on the new platforms. In the near future, the ideal structured curriculum should cover even broader skills, enabling new surgeons to work across all different platforms. However, predicting how training will be affected by an increasing number of robotic systems is difficult.
